# Anatomical Grading for Metabolic Activity of Brown Adipose Tissue

**DOI:** 10.1371/journal.pone.0149458

**Published:** 2016-02-22

**Authors:** Anton S. Becker, Hannes W. Nagel, Christian Wolfrum, Irene A. Burger

**Affiliations:** 1 Department of Nuclear Medicine, University Hospital of Zurich, Zurich, Switzerland; 2 Department of Diagnostic and Interventional Radiology, University Hospital of Zurich, Zurich, Switzerland; 3 Department of Health Sciences and Technology, ETH Zurich, Zurich, Switzerland; St. Joseph's Hospital and Medical Center, UNITED STATES

## Abstract

**Background:**

Recent advances in obesity research suggest that BAT activity, or absence thereof, may be an important factor in the growing epidemic of obesity and its manifold complications. It is thus important to assess larger populations for BAT-activating and deactivating factors. ^18^FDG-PET/CT is the standard method to detect and quantify metabolic BAT activity, however, the manual measurement is not suitable for large studies due to its time-consuming nature and poor reproducibility across different software and devices.

**Methodology/Main Findings:**

In a retrospective study, 1060 consecutive scans of 1031 patients receiving a diagnostic ^18^FDG-PET/CT were examined for the presence of active BAT. Patients were classified according to a 3-tier system (supraclavicular, mediastinal, infradiaphragmatic) depending on the anatomical location of their active BAT depots, with the most caudal location being the decisive factor. The metabolic parameters (maximum activity, total volume and total glycolysis) were measured on a standard PET/CT workstation. Mean age of the population was 60±14.6y. 41.61% of patients were female. Metabolically active BAT was found in 53 patients (5.1%). Female, younger and leaner patients tended to have more active BAT, higher metabolic activity and more caudally active BAT. In total, 15 patients showed only supraclavicular, 27 additional mediastinal, and 11 infradiaphragmal activity. Interestingly, the activation of BAT always followed a cranio-caudal gradient. This anatomical pattern correlated with age and BMI as well as with all metabolic parameters, including maximum and total glycolysis (p<0.001).

**Conclusion:**

Based on our data we propose a simple method to grade or quantify the degree of BAT amount/activity in patients based on the most caudally activated depot. As new modalities for BAT visualization may arise in the future, this system would allow direct comparability with other modalities, in contrary to the PET-metrics, which are restricted to ^18^FDG-PET/CT.

## Introduction

Obesity has expanded as an epidemic in the developed and developing world, with the WHO estimating an approximate of 600 million people being obese and more than 1.9 billion adults belonging to the overweight category [1–4]. It is defined as the “abnormal or excessive fat accumulation that may impair health” [1] due to long-term positive energy balance, usually caused by reduced levels of physical activity/exercise and/or high consumption of energy-dense foods. Obesity has been associated with several co-morbidities including type II diabetes, cardiovascular disease, musculoskeletal disorders such as osteoarthritis and some types of cancer [5]. Therefore, prevention and treatment of obesity is of high importance, however lifestyle changes exert only modest results and do not seem to be successful in the long term [6]. Additionally, pharmacological approaches are limited and ineffective. In fact, several anti-obesity drugs have been developed, but not been approved due to severe side effects [7]. It is thus important that novel, pharmacological and non-pharmacological anti-obesity treatments are developed in order to reduce the burden of obesity.

The adipose organ (reviewed in detail by Rosen et al. [8]) is a multi-depot endocrine organ that can be macroscopically divided into two different types: the white and the brown adipose tissue, based on the predominant form of adipocyte. White adipose tissue (WAT) constitutes the major storage depot of excess energy in the form of triacylglycerols (TGs) in the liposomes of white adipocytes as well as an important endocrine organ. On the contrary, the main function of brown adipose tissue (BAT) is non-shivering thermogenesis (i.e. heat production) in brown adipocytes. The protein responsible for this particular function of brown adipose tissue–constituting at the same time the only brown adipose tissue specific marker in humans–is uncoupling protein 1 (UCP1). UCP1 is located in the inner mitochondrial membrane and dissipates the proton (H^+^) gradient formed as a result of oxidative phosphorylation, which is normally used for phosphorylation of ADP, thus uncoupling mitochondrial respiration from ATP production. The mitochondrion is thus running “out of gear” and the energy dissipates in heat.

A third form of fat cell, the brite (“brown-in-white”) or beige adipocyte has been characterized microscopically and is still being studied extensively in vitro and in vivo. [9]. To date, brown fat is indistinguishable from brite fat in FDG-PET/CT as no activity threshold or specific anatomic locations in humans have been established. In the following sections we shall henceforth use BAT as a synonymous abbreviation for brown and brite fat.

Many mammals like the hedgehog or mouse use BAT for their thermostasis during hibernation [10]. In humans, it was long believed that only neonates and young children have metabolically active BAT. Even though in autopsy series BAT was found in adults [11], the evidence in favor of BAT being metabolically active in adults [12] was largely ignored and the dogma persisted, that BAT rapidly loses its function after childhood. Thanks to the ability of ^18^FDG-PET/CT to match glycolysis with exact anatomical location, metabolic activity in BAT in adults was first visualized by Hany et al. in 2002 [13].

Since then in depth analyses in adult human studies were performed [14–16], and there has been a growing interest in brown adipose tissue as a novel therapeutic approach to activate energy dissipation in BAT in order to aid and accelerate weight-loss. In the last few years a host of human studies have been reported demonstrating the correlation of (absence of) active BAT with multiple parameters linked to obesity and the development of metabolic disorders. It has been shown that gender, age, BMI as well as central obesity have an influence on the presence of active BAT [17]. Furthermore, in experimental studies, several factors have been discovered that either activate or deactivate BAT. For example, it was shown that physical application of cold [18] or oral intake of bile acids [19] or selective β_3_-agonists [20] can activate brown fat [15] whereas propranolol, a non-selective β-blocker, pharmacologically blocks the activation of BAT [21].

From the first description of active BAT in adult humans in 2002 [13], ^18^FDG-PET/CT has played not only a crucial role in subsequent studies [14–17, 22–25], but has become the de-facto “gold standard” for detecting active BAT. Indeed, ^18^FDG-PET/CT is ideal to identify new and to discern strongly from weakly activating or inhibiting factors and to quantify the intensity of BAT activation. Through the combination of PET and CT imaging metabolic activity can not only be precisely matched to the anatomic location, but also accurately measured in individual regions of the 3D volumetric dataset. Albeit accurate, this manual measurement is also very time-consuming and results may not be comparable across different devices. It is thus unsuitable for large, prospective, multi-center trials.

In the present retrospective study we examined the influence of known BAT-activating factors in relation to glucose metabolism (i.e. intensity of activation) using various quantitative PET metrics, as well as anatomic location and distribution patterns of the activated depots to find a simple and robust method for quantification of BAT activity.

## Materials and Methods

### Patient Population

This study was approved by the local ethics committee of the University of Zürich, who waived the need for informed consent. All patients referred for a diagnostic ^18^FDG-PET/CT from November 2014 to January 2015 at the University Hospital of Zürich were included. No patients were excluded.

Pre-scan fasting blood glucose level of all patients with active BAT was obtained post hoc from the patient records.

### Image Acquisition

All patients were scanned on a dedicated PET/CT machine (GE Healthcare DSTX, 16-or 64-slices CT, 7–8 frames, frame time 1.5 or 2 minutes). Fasting for 6 hours prior to the study, including sweetened beverages and chewing gum, was mandatory. Blood glucose was measured prior to the FDG-injection and had to be below 7 g/l [26]. Patients received approximately 4 MBq ^18^F-FDG per kilogram bodyweight, followed by a 60 ± 5 min. uptake period. Afterwards, a low-dose, attenuation correction CT scan was acquired (100–120 kV, approx. 80 mA), followed by the PET scan from mid-thigh to the vertex of the skull.

### Image Analysis

Images were analyzed on state-of-the-art dedicated workstation for PET/CT image reading (AW version 4.6, GE Healthcare DSTX) by one reader dually specialized in radiology and nuclear medicine. The activity of BAT was measured in standardized uptake values (SUV [g/ml]), a measure commonly used in PET/CT in the clinical routine.

All patients were screened for the presence of active BAT using a 3D maximum intensity projection (MIP) image of the PET scans. In a MIP image, the volumetric image data is reduced to a 2D planar projection, and only the voxel with the highest value (i.e. maximum metabolic activity) is used for the pixel of the projected image. The PET and CT images were subsequently electronically co-registered and digitally fused, with a colorized PET image overlaying the CT image. The color scale (“Perfusion”, GE Healthcare DSTX) was set between SUV 0–6 g/ml for visual analysis. In all positive or ambiguous cases, the fused PET/CT series was used for a definitive decision. BAT was considered metabolically active, when a metabolically active area in the MIP image could be appreciated, that subsequently matched to a fat-density area in the CT image (-250 to -50 Hounsfield Units).

Subsequently, in all patients with active BAT the maximum and mean SUV (SUV_max_, SUV_mean_), the total fat gylcolysis (TFG; counterpart to the normally used PET-metric “Total lesion glycolysis TLG”) and metabolically active fat volume (MFV; counterpart to “Metabolic tumor volume MTV”) of the BAT were measured. The cut-off for TFG and MFV was set at 2.5 g/ml. In cases with a SUV_max_ below 2.5, the background signal in white fat was measured and used as a cut-off. Patients were classified according to a 3-tier system depending on the anatomical location of their active BAT.

### Statistical Methods

Statistical analysis was done with R version 3.2.1 (R Core Team (2015). R: A language and environment for statistical computing. R Foundation for Statistical Computing, Vienna, Austria. URL http://www.R-project.org/). Graphs/plots were produced with the ggplot2 package [27]. Due to the small proportion of BAT-positive patients compared to BAT-negative patients, density plots were used for the distribution of age and BMI as opposed to conventional histograms. The remaining tests were performed using native R functions. We used descriptive analysis (mean, standard deviation, median) for all relevant continuous parameters, regression analysis with a general linear model for anatomical distribution to BMI, temperature or age, as well as Student's t-test for continuous variables and Fisher's exact test (Presence of BAT—age / BMI / sex / temperature, sex—metabolic activity / anatomical distribution / blood glucose). A p-value of < 0.05 was considered statistically significant; Bonferroni correction was used where appropriate.

## Results

### Identification of BAT positive patients

To identify patients with positive BAT signatures we retrospectively analyzed 1060 scans of 1031 patients, scanned from November 2014 to January 2015 at our institution. The mean FDG activity used in the PET-CT scan was 4 MBq per kg bodyweight, mean time between application and scan was 61 ± 9 minutes. The mean age of the analyzed individuals was 60 years (median 62 y, range 9–97 y, ± 14.6 y). Males accounted for 59.4% of the population, female patients for 40.6%, respectively. The mean BMI of the studied subjects was 24.9 ± 4.8 kg/m^2^. The female subjects included in the study were slightly but significantly younger (3.4 years) and had a lower BMI (4.3 points) than males (p < 0.001). To correlate BAT activity with outside temperature, we acquired the average temperature for the day of the scan from a commercial provider (MeteoGroup Ltd., Berlin, Germany). Their closest weather station is located about 800 m from the hospital, providing a good approximation of the daily temperatures at the site of the examination. The mean temperature was 3.9 ± 3.6°C, with a maximum temperature of 11.2°C during the three-month-period.

In the FDG-PET scan, BAT with a metabolic activity (SUV_max_) above 2.5 g/ml was considered as positive. To avoid false positives of other activity (e.g. muscles, lymph nodes), the precise anatomic location of the metabolic activity was always verified on the digitally fused PET/CT slices.

**Fig 1:**
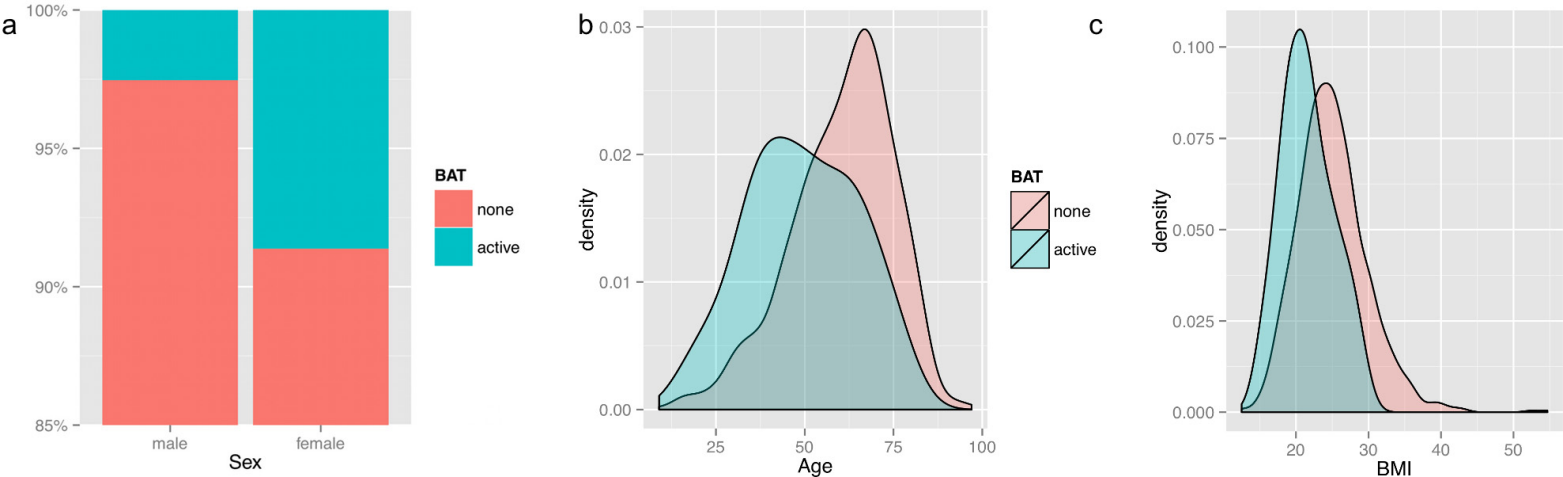
Differences between the two cohorts. **a) Sex difference**: Stacked bar plot depicting the top 15% percent of the overall population, showing the higher incidence (OR = 3.62) of positive BAT in females compared to males. **b) Age** and **c) BMI difference of the BAT-positive cohort.** Density plots showing the shift of age and BMI from the overall population to the BAT-positive cohort (blue), who were found to be significantly younger and lighter.

Metabolically active BAT was found in 53 patients (5.1%) and there were no patients with active BAT on more than one scan in the given time period. Mean age in BAT-positive patients was 49.32, (median 49, range 17–77). We identified 17 male and 37 female subjects with positive BAT signature and as reported previously [14, 16, 17, 24] female patients were significantly more likely to show metabolically active BAT than male patients (estimated odds ratio of 3.62, p < 0.001) independent of age (p = 0.24) (Fig 1A). Similarly, we observed a significant shift in age (Fig 1B) and BMI (Fig 1C) distribution, indicating that BAT activity is inversely correlated with both parameters. Similarly to the whole population, female BAT-positive patients showed a lower BMI compared to males (19.6 vs. 23.66, p = 0.02), and we did not observe any difference in mean daily temperature for males and females (p>0.6). The details of the cohort are summarized in Table 1.

**Table 1:** Cohort details.

Category	Overall	BAT-Positive	BAT-Negative
N	1060	53	1007
Sex (Male/Female)	631 / 429	16 / 37 †	615 / 392
Age (Years)	60.04±14.65	49.32±15.54 †	60.6±14.39
Height (m)	1.71±0.09	1.67±0.07 †	1.71±0.09
Body Weight (kg)	72.68±16.19	60.51±12.35 †	73.32±16.12
BMI (kg/m^2^)	25.16±6.23	20.86±4.77 †	25.38±6.21
Temperature (°C)	3.88±3.58	3.07±3.43	3.92±3.58

Indicated are the numbers of cases and continuous parameters from the whole population as well as separated by BAT-activity as means ± SD. Highly significant differences, i.e. p < 0.001, in the BAT-positive cohort compared to the overall population are marked with a dagger sign (†)

### Patterns and Activity of BAT

**Fig 2:**
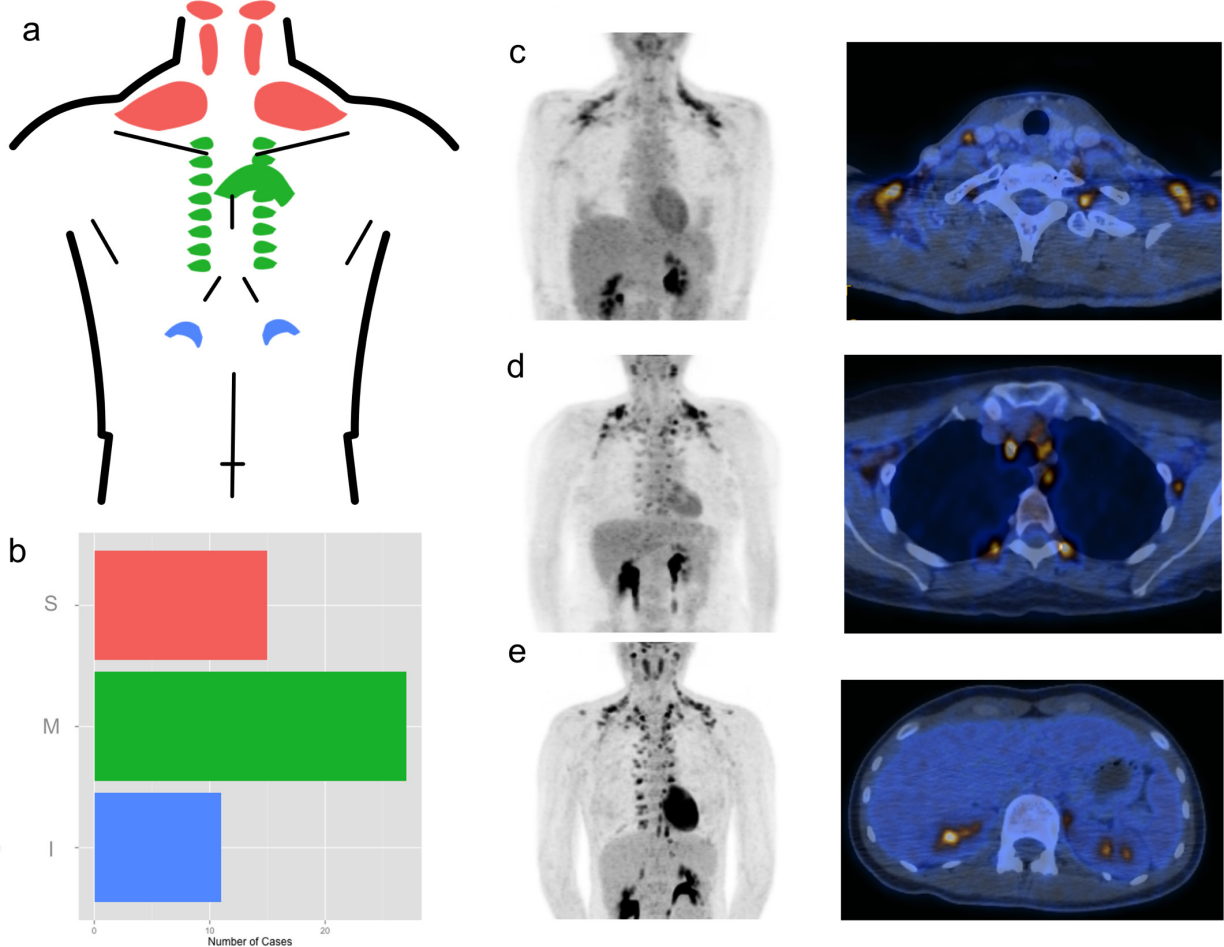
Morphologic Patterns of BAT. **a)** Schematic drawing of the different depots, some of which were summarized as indicated by the coloration into the three categories **c)** supraclavicular **d)** mediastinal and **e)** infradiaphragmatic. **b)** Showing the total number of BAT-positive subjects grouped by the most caudally active depot of either supraclavicular (including axillary and nuchal), mediastinal or infadiaphragmal BAT. **c-e)** Coronal MIP PET images (left) and fused PET/CT slices (right) of representative subjects in the according category. All pictures also show variable glucose uptake of the liver, intestines and myocardium as well as strong emissions from the renal pelvis, the latter being concentrated ^18^FDG breakdown products excreted through the urine. This can be appreciated in the fused **d)** image, where the true BAT activity is located in a fat depot on the patient’s left side (right side on the picture) adjacent to the first lumbar vertebra.

In our 53 BAT-positive subjects we observed an average SUV_max_ of 7.8 ± 4.4, an average SUV_mean_ of 3.12 ± 0.58 and average TFG of 12782 ± 98172 and an average MFV of 76.2 ± 101.3 cm^3^ (± SD), respectively. From our clinical experience, we noticed an activation of BAT in distinct anatomical locations. We thus classified the distribution of active BAT according to a three-tier system, with three easy-to-identify main depots: Supraclavicular (including axillary and nuchal), Mediastinal and Infadiaphragmal (Fig 2).

**Fig 3:**
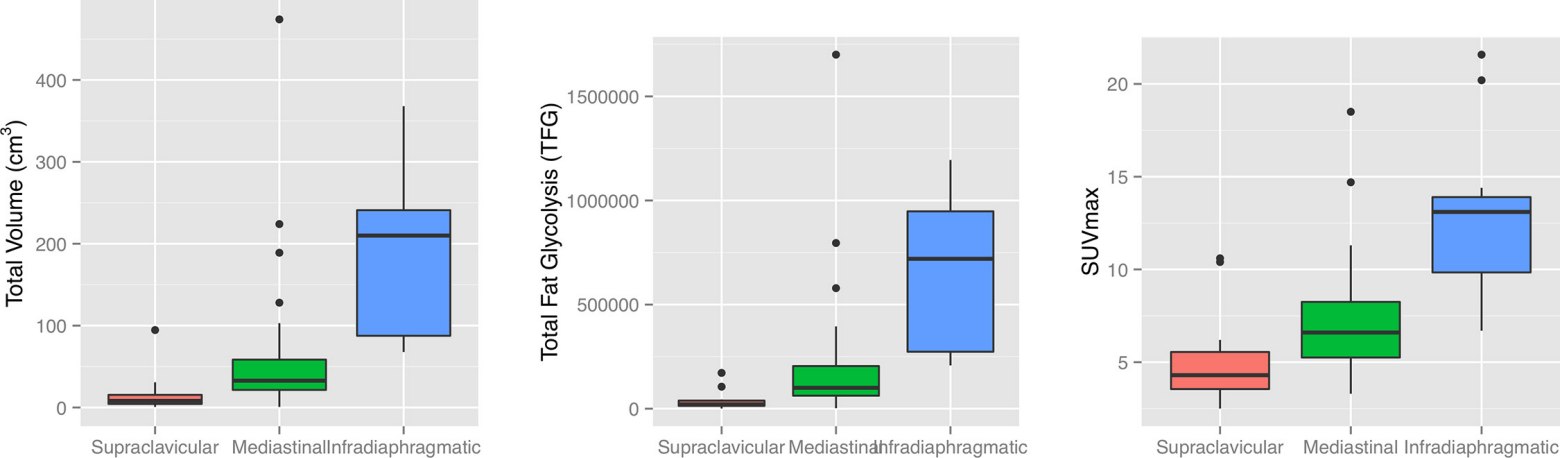
Quantification of glycolysis in the different depots. A clear increase of all three metabolic PET-parameters MFV, TFG and SUV_max_, from the supraclavicular depots to the more caudally located infradiaphragmatic depots can be appreciated.

Of the 53 patients with active BAT, 15 showed only supraclavicular, 27 additional mediastinal, and 11 infradiaphragmal activity. (Fig 2B). The activation of BAT always followed a strictly cranio-caudal gradient, i.e. no patient had active BAT in the abdomen without active BAT in the mediastinum and supraclavicular area, and there were no cases with active BAT in the mediastinum without supraclavicular activation as well. Furthermore, BAT total volume, total fat glycolysis and SUV_max_ correlated significantly with the localization of BAT (Fig 3), suggesting that the further caudally the active BAT depots are observed, the more BAT gets recruited and the higher is the maximum BAT activity as well as the total BAT glycolysis.

**Fig 4:**
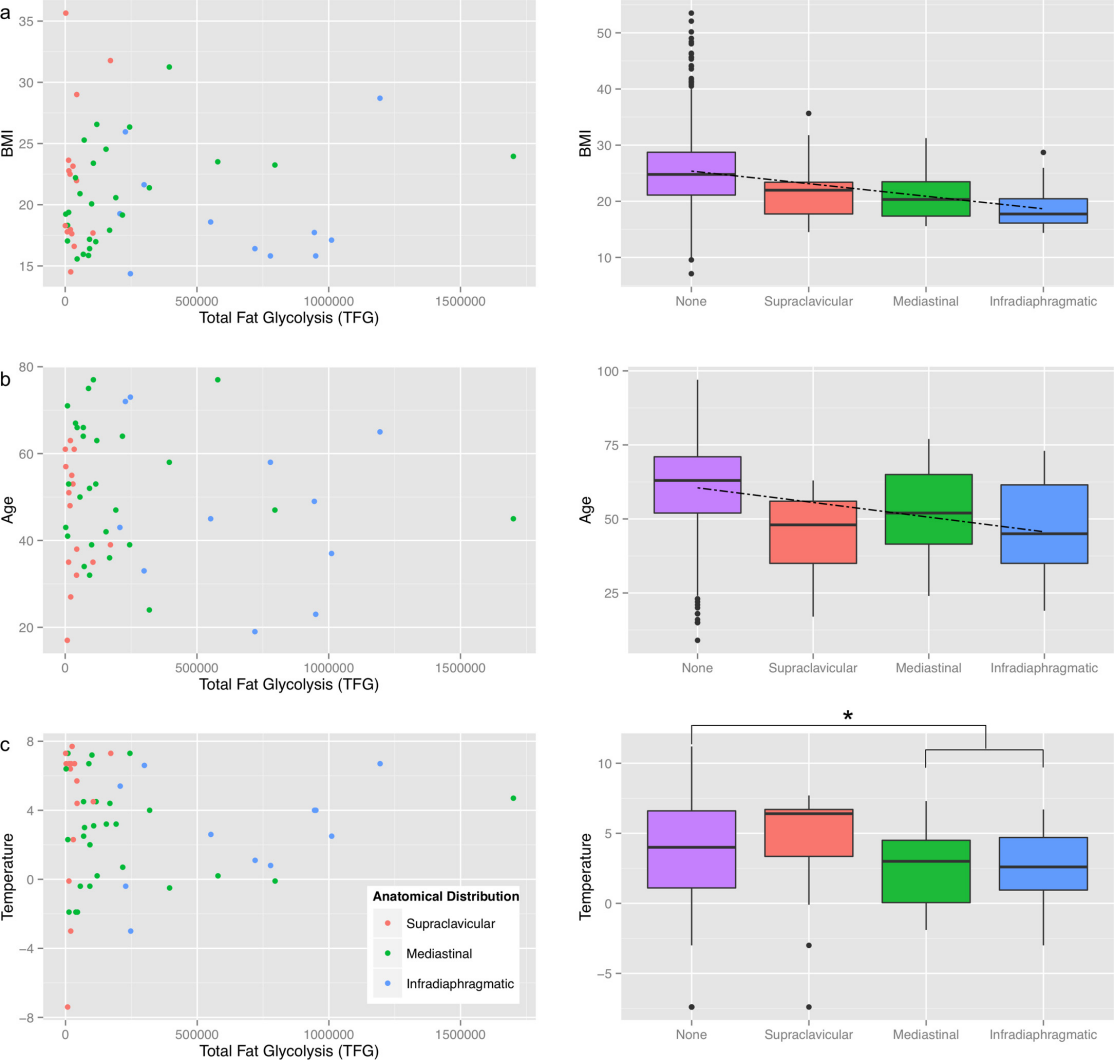
Differences in BAT activation between individuals and categories. The first column depicts the total fat glycolysis (TFG) in relation to BMI **(a)**, age **(b)** and external temperature **(c)**. As can be guessed by the visual impression of the scatter plots, no clear correlation between either of the factors and the TFG could be established. In the second column however, the same data is shown and instead of using the metabolic activity, it is simply ordered by the morphological type of activation, showing clearly more activated depots with lower BMI, age and temperature. The dashed lines in (a) and (b) represent the significant linear correlation (p > 0.05), the asterisk (*) in (c) marks the only statistically significant difference (p<0.0125) in absence of a linear correlation.

Interestingly, these findings also correlated with different metabolic parameters as indicated in Fig 4. For example we could show that not only BMI correlated with BAT localization (p < 0.05; Fig 4A), a similar correlation was observed for age (p < 0.01; Fig 4B) suggesting that younger and leaner patients tended to have more active BAT and showed more BAT in the infradiaphragmatic region. In contrast to BMI and age, no correlation with the external temperature could be established (p = 0.17; Fig 4C and Table 2). However, the difference of the mean temperature between no activation, and mediastinal and infadiaphragmatic activation combined was significant (p<0.0125).

**Table 2:** Morphological differences in BAT activity.

Pattern	Supraclavicular	Mediastinal	Infradiaphragmatic
N	15	27	11
Sex (Male/Female)	7 / 8	8 / 19	1 / 10
Age (Years)	44.8±14.02	52.78±14.8	47±18.51
Height (m)	1.68±0.08	1.67±0.06	1.67±0.07
Body Weight (kg)	61.13±16.16	61.26±10.42	57.82±11.63
BMI (kg/m^2^)	22.06±6.01	20.85±3.99	19.22±4.5
Temperature (°C)	4.13±4.41	2.6±2.95	2.75±3
Fasting blood glucose (mmol/l)	4.9±0.6	5.1±0.8	4.9±0.6
SUV_max_	5.01±2.47	7.22±3.39	12.93±4.61
TFG	36595±45133	217268±347291	648555±359665
MFV (cm^3^)	16.26±23.45	65.71±97.83	183.86±96.41

Shown are the differences in demographic data and mean daily temperature (upper part), as well as the metabolic parameters (lower part), between the cohorts with the different BAT-activation patterns. All metabolic PET-parameters showed a significant correlation with the anatomical activation pattern (p < 0.001).

At very cold temperatures (< -2°C), only three cases of active BAT were observed, two with supraclavicular type, i.e. “weak” activation (SUV_max_ 3.5, 2.9), and one with supraclavicular, mediastinal and infradiaphragmatic, i.e. “strong” activation (SUV_max_ 10.9). Within the different morphological categories, no significant correlations for age, BMI or temperature could be found, as can be appreciated in the scatterplots (Fig 4A and 4B): While the different colors (= anatomic locations) form notable clusters, within each color/location no pattern can be found.

Fasting blood glucose levels were not significantly different between the different morphological categories. There was a tendency for the fasting blood glucose to correlate weakly with the BMI, though it did not reach statistical significance in our sample (p = 0.09).

## Discussion

The propagating epidemic of obesity in industrialized as well as developing nations, and all of the entailed complications (diabetes, arterial hypertension and cancer, to name only a few [6]), have led to enormous efforts in clinical and laboratory research in the past decades to understand the function of different fat tissues in its pathogenesis [28]. The description of metabolically active BAT in 2002 and the subsequent studies demonstrating the presence of brown fat by molecular analysis [13–16, 18] have sparked a wide interest in this tissue. Although semi-automatic measurement of FDG-activity can be done in cancer patients [29], in the case of BAT it is very prone to false positive measurements, since physiologic brain or heart activity or malignant tumor activity could not be distinguished from BAT. Thus, the current method of measuring BAT activity implicates manually placing regions of interest in the PET/CT scan (SUV_max_), and manually segmenting the picture (TFG, MFV) by cropping other metabolically active tissues like the myocardium, liver or skeletal muscles. This process is neither suitable for larger studies due to its tedious, time-consuming nature, nor for multi-center trials, due to the lack of harmonized PET quantification due to institution and scanner specific imaging and reconstruction algorithms [30], as well as potential inter-operator differences in the manual segmentation. Although different, easy-to-identify anatomical locations of BAT depots have been described, there has yet to be any attempt to use them for a simpler grading system of BAT activation.

Our inclusion threshold SUV of 3 g/ml was slightly higher than in most previous studies (SUV of 1–2 g/ml) to reduce the possibility of false positives. In our population, we found a BAT incidence of 5.1%, which is in concordance with previously reported studies (e.g. 5.4% from Cypess et al. [14]). Furthermore, in line with previous studies [15–18, 22, 24, 31, 32] we identified significantly more females, as well as younger and leaner (i.e. lower BMI) patients in the BAT positive cohort than in the whole population.

In our set of analysis we did not observe a significant difference in the daily temperature between the BAT positive and negative patients. This is most probably due to the fact that we deliberately chose the three cold winter months November to January with a mean temperature lower than 4°C and a maximum of 11.2°C for our retrospective study in order to obtain a higher prevalence in the studied population, as previously reported [14].

In previous PET and autopsy studies, in total 18 anatomical locations of active BAT have been reported [11, 33]. Their categorization is based on the depth of the depot, with the subcutaneous ones representing the most superficial ones, and the vicinity to relevant anatomical structures like vessels, viscus or parenchymatous organs.

For practical purposes, we summarized the many different depots in three categories: Supraclavicular, mediastinal and infradiaphragmatic. The names describe the visually most accentuated depot: The supraclavicular category contains the axillary depot, which is strictly anatomically speaking located below the clavicles (infraclavicular). We still used the name “supraclavicular” in spite of a more accurate label like “supramammillary extrathoracal” because it is simpler and commonly used in the existing literature. While a finer graduation is definitely possible, e.g. dividing the nuchal from the supraclavicular depot, more categories (and thus less cases per category) make it more difficult to obtain an applicable grading system. Furthermore, some of the described depots such as the subcutaneous depot in the anterior abdominal wall, are known to be inactive after the first decade of life [11].

A large advantage of an anatomy-based grading system is the direct applicability to other imaging modalities. For example, we would be able to compare study data across different PET-tracers in case a new, more specific PET tracer would replace the fairly unspecific ^18^FDG as the standard in detecting BAT in the future. This direct comparison is not possible with the standard PET-metrics. The SUV and its derived SUV_max_, TFG and MFV are always tracer-specific, in addition to the problems in precise quantification discussed above.

Another advantage is the abovementioned timesaving aspect of being able to directly grade BAT-activity in the standard picture archiving and communication (PACS) system. Whole-body PET/CT-scans consist of large amounts of image data, the transfer of which to a separate workstation alone will typically take between 5 and 10 minutes at current network speeds. Manual SUV measurement would generally take at least another 5 minutes, semi-automatic measurement by specifying a SUV threshold [29] is substantially faster but also very prone to false positive activity eg. in the myocardium, liver or skeletal muscle. In contrast, retrieving the images in the PACS software and anatomical grading can be achieved in under one minute.

The obvious correlation between more depots activated and more metabolically active volume could easily be established. Furthermore, the total glycolysis as well as maximum activity also increased, the more (i.e. further caudally) depots were activated. This is an interesting finding, which could be explained by several mechanisms. On the one hand it is possible that the depots differ in β_3_ adrenergic receptors density [20], thus altering their propensity to be activated. Furthermore, it is possible that either the innervation varies between individuals or general differences in sympathetic output patterns lead to the different activation patterns.

Since this is a retrospective study in a collective of mostly non-healthy patients, further prospective trials in healthy subjects are warranted to investigate these questions. Moreover, in a prospective trial the correlation with more biomarkers for example in blood or small tissue samples would be possible.

To summarize, our results confirm that BAT activation is observed especially in females and in younger, leaner patients. Furthermore they also confirmed the hypothesis that the sequential cranio-caudal (“top-down”) activation correlates with all the quantitative PET parameters for BAT activity, and thus might be used as a simple and robust grading system for BAT activation instead of manually or semi-automatically performed, quantitative PET measurements.
